# BRD4-targeting PROTAC as a unique tool to study biomolecular condensates

**DOI:** 10.1038/s41421-023-00544-0

**Published:** 2023-05-09

**Authors:** Yi Shi, Yuan Liao, Qianlong Liu, Zhihao Ni, Zhenzhen Zhang, Minglei Shi, Pilong Li, Haitao Li, Yu Rao

**Affiliations:** 1 MOE Key Laboratory of Protein Sciences, School of Pharmaceutical Sciences, MOE Key Laboratory of Bioorganic Phosphorus Chemistry & Chemical Biology, Tsinghua University, Beijing, China; 2 Tsinghua-Peking Joint Center for Life Sciences, Tsinghua University, Beijing, China; 3 Ministry of Education Key Laboratory of Protein Sciences, Beijing Advanced Innovation Center for Structural Biology, Beijing Frontier Research Center for Biological Structure, Department of Basic Medical Sciences, School of Medicine, Tsinghua University, Beijing, China; 4 Peking University-Tsinghua University-National Institute of Biological Sciences Joint Graduate Program, Beijing, China; 5 MOE Key Laboratory of Bioinformatics, Bioinformatics Division and Center for Synthetic & Systems Biology, BNRist, School of Medicine, Tsinghua University, Beijing, China; 6 Beijing Advanced Innovation Center for Structural Biology, Tsinghua-Peking Joint Center for Life Sciences, School of Life Sciences, Tsinghua University, Beijing, China

**Keywords:** Transcriptional regulatory elements, Super-resolution microscopy

## Abstract

Biomolecular condensates play key roles in various biological processes. However, specific condensation modulators are currently lacking. PROTAC is a new technology that can use small molecules to degrade target proteins specifically. PROTAC molecules are expected to regulate biomolecular condensates dynamically by degrading/recovering key molecules in biomolecular condensates. In this study, we employed a BRD4-targeting PROTAC molecule to regulate the super-enhancer (SE) condensate and monitored the changes of SE condensate under PROTAC treatment using live-cell imaging and high-throughput sequencing technologies. As a result, we found that BRD4-targeting PROTACs can significantly reduce the BRD4 condensates, and we established a quantitative method for tracking BRD4 condensates by PROTAC and cellular imaging. Surprisingly and encouragingly, BRD4 condensates were observed to preferentially form and play specialized roles in biological process regulation for the first time. Additionally, BRD4 PROTAC makes it possible to observe the dynamics of other condensate components under the continued disruption of BRD4 condensates. Together, these results shed new light on research methods for liquid-liquid phase separation (LLPS), and specifically demonstrate that PROTAC presents a powerful and distinctive tool for the study of biomolecular condensates.

## Introduction

Biological macromolecules, such as proteins and RNAs, can form biomolecular condensates by liquid–liquid phase separation (LLPS) and play key roles in various biological processes including RNA transcription and translation, and cell signaling regulation^1–4^. Some studies have shown that abnormal phase separation may lead to a series of downstream gene dysfunction causing diseases^3,5,6^. A large number of transcription factors, enzymes, and splicing complexes are involved in the transcription process, which can form biological condensates to facilitate this process^7–13^. Models for transcription regulation through LLPS are rapidly evolving^14,15^. Although large progress has been made in building these models, many details remain unclear^16^. An important reason is the lack of effective tools to regulate biomolecular condensates. Although 1,6-HD is thought to disrupt hydrophobic interactions and thereby disrupt LLPS, the disruptive effects of 1,6-HD are nonspecific, resulting in extensive damage to intracellular biomolecular condensates and indiscriminate cell death^17^. Therefore, targeting key genes within specific biomolecular condensates is a feasible idea. CRISPR-Cas9, RNA interference (RNAi) and chemical inhibitors are commonly used tools targeting proteins, however, they have their limitations in studying phase separation in vivo and in vitro. First, gene-editing tools are irreversible to DNA modifications, limiting our observation of the state of the encoded protein. Second, due to the long onset time of RNAi (at least 48 h), RNAi is not a suitable tool for observing a highly dynamic phase separation process^18^. Moreover, these genetic tools, including ectopic expression of light-sensitive proteins to control LLPS-related gene expression^19^, still fail to directly interfere with LLPS on wild-type cells under physiological conditions. Third, small molecule inhibitors can induce occupancy-driven inhibition of proteins and partially affect condensate formation through binding the active/enzymatic sites of proteins^10,20–22^. However, most of the condensate formation is induced by inactive/non-enzymatic or scaffold domains. In addition, small molecule inhibitors often cause transient upregulation of the target protein, resulting in a higher cell dosing requirement^23^.

In summary, LLPS is a highly dynamic and reversible biological process under certain conditions. How to rapidly and reversibly regulate LLPS? How to study the interaction mechanism of key proteins in LLPS? New tools are urgently needed to facilitate addressing these issues^24^.

Proteolysis-targeting Chimera (PROTAC), is an emerging technology that induces degradation of target protein through the ubiquitin-proteasome system. Unlike small molecule inhibitors that occupy the active pocket of target proteins, PROTAC molecules directly degrade target proteins in a catalytic cycle^25,26^. PROTAC can regulate target proteins rapidly, reversibly, efficiently, and conveniently, so it is expected to provide a new strategy for the dynamic regulation of target proteins in vivo and in vitro^27^. The formation and dynamics of phase separation are highly dependent on the concentration of biomacromolecules^28^, so adjusting the concentration of biomacromolecules can modulate phase separation. Given this, PROTAC could serve as a useful and unique tool to degrade the target proteins in time-dependent and dose-dependent manners, and the degradation of target proteins is reversible.

Our research focuses on the vital epigenetic protein BRD4 involved in transcriptional regulation. BRD4, a member of the Bromodomain and Extraterminal (BET) protein family, is both a key component of super-enhancer (SE) and a reader of histone acetylation and regulates important biological processes such as DNA damage and chromatin remodeling^29,30^. To date, BRD4 is a popular anticancer drug development target, because it assembles at SEs to form condensates and plays a critical role in the regulation of oncogene expression^31^. Cancer cells utilize SEs to form condensates for regulating cell proliferation and survival through critical oncogenes^32,33^. The introduction of PROTAC in LLPS would allow a better understanding of the dynamic mechanism of SEs assembly and other transcriptional components to promote oncogene expression.

**Fig. 1:**
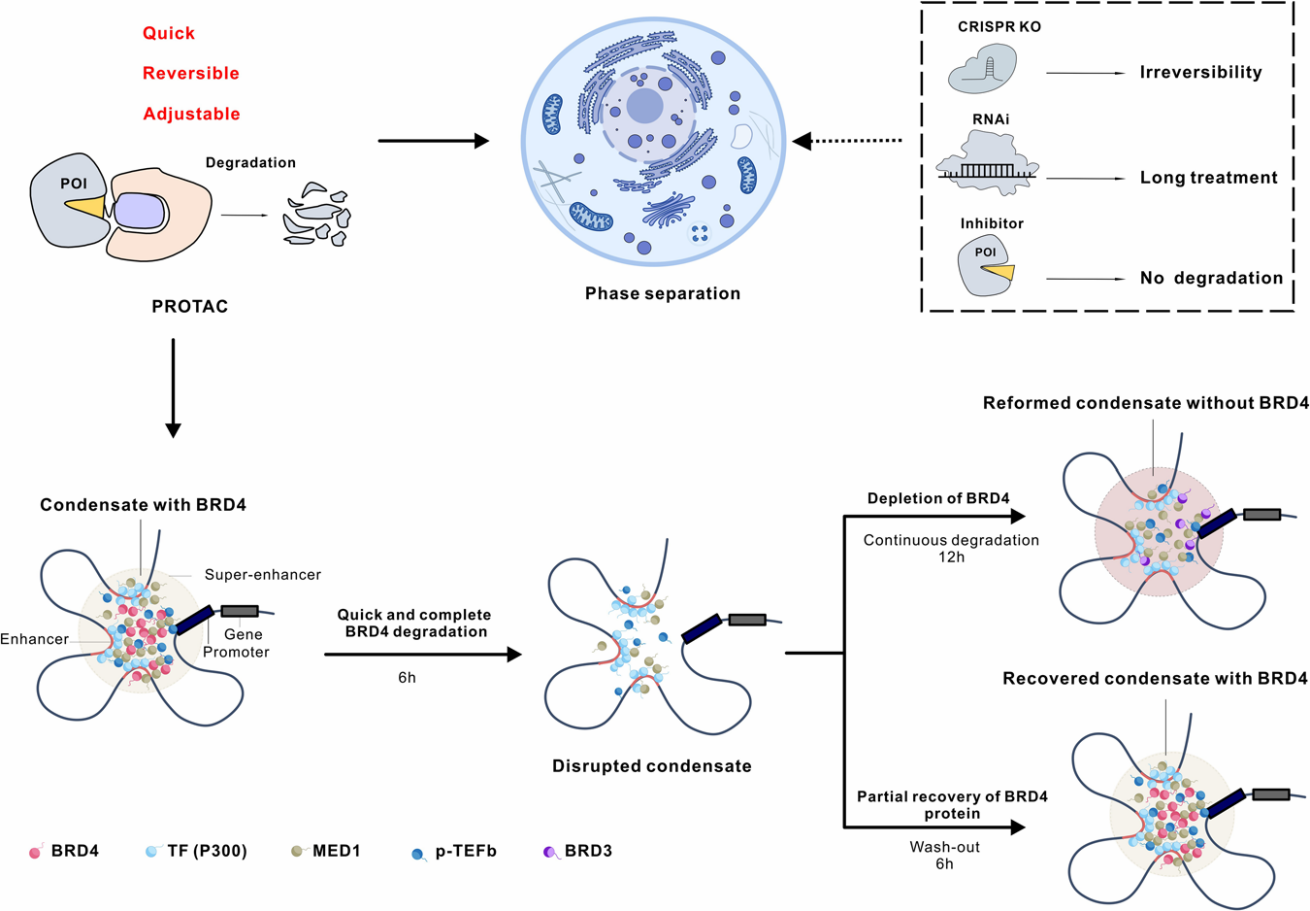
The schematic diagram of phase separation studies using PROTAC. PROTAC is believed to offer a new method for dynamic regulation in vivo and in vitro since it can regulate target proteins quickly, reversibly, effectively, and conveniently. The quick and reversible nature of PROTACs enables dynamic monitoring over a shorter time and allows us to detect phenomena that cannot be observed by genetic techniques like CRISPR-Cas9 or RNAi without being impacted by compensating effects following genetic alteration.

Here, we used PROTAC molecules to study the components of BRD4 condensates and find the key factors driving biological macromolecule condensates at SEs (Fig. 1). At the cellular level, rapid and efficient degradation of BRD4 protein was carried out by PROTACs, and the changes of target protein condensates were tracked by immunofluorescence. This study established a “PROTAC-target protein-LLPS” method to explore the dynamic changes and mechanisms of BRD4 condensates at the SE site. Our work represents the first application of the PROTAC in LLPS. We believe that it can provide new insights into LLPS and facilitate further research on LLPS in cancer pathology and related drug development.

## Result

### Establishment of the “PROTAC-target protein-LLPS” method

**Fig. 2:**
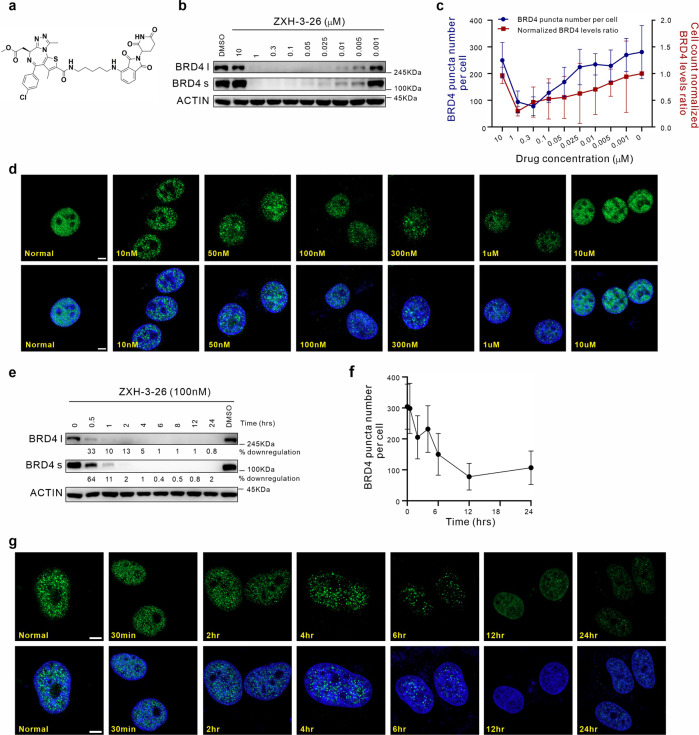
Establishment of the “PROTAC-target protein-LLPS” method. **a** Chemical structure of ZXH-3-26. **b** Western blot analysis of BRD4 in HeLa cells treated with different concentrations of ZXH-3-26 for 6 h. **c** Joint analysis of BRD4 protein levels and condensate numbers with different concertation of ZXH-3-26 treatment (*n* = 30–35 cells). **d** Immunofluorescence was performed to track BRD4 condensates after different concentrations of ZXH-3-26 treatment for 6 h. Scale bars, 5 μM. **e** BRD4 was detected by western blot analysis at different time points after ZXH-3-26 treatment at 100 nM concentration. **f** Each time point sample was quantified for the average BRD4 condensate number by adding 100 nM ZXH-3-26. *n* = 30–35 cells per time point. **g** Immunofluorescence detection of BRD4 condensates at different time points treated with 100 nM ZXH-3-26. Scale bars, 5 μM.

To validate the effect of PROTAC on the condensates of the target protein, we applied a candidate PROTAC molecule named ZXH-3-26 (Fig. 2a)^34,35^, which can selectively degrade endogenous BRD4 in dose-dependent and time-dependent manners, but not BRD2 and BRD3 (Supplementary Fig. S1a–c). BRD4 has two main transcripts, long (BRD4 l) and short (BRD4 s) isoforms^30^. ZXH-3-26 degraded both long and short isoforms simultaneously. (Fig. 2b, e). ZXH-3-26 did not induce apoptosis even after 48 h treatment at 100 nM (Supplementary Fig. S1d, e), unlike the case of 1,6-HD, the compound that breaks hydrophobic interactions to disturb condensates, resulting in cell death^17^. Therefore, we selected ZXH-3-26 at a concentration of 100 nM for most subsequent experiments.

Current criteria for determining biomolecular condensates are: (1) forming a spherical structure; (2) being able to fuse; (3) being able to recover from fluorescent bleaching using fluorescence recovery after photobleaching (FRAP) technology^36^. However, these criteria still fail to determine the threshold for LLPS-associated target proteins in living cells in an endogenous state. Here, we used ZXH-3-26 to degrade endogenous BRD4 proteins and tracked their condensation changes using cell imaging techniques. To this end, we assessed protein degradation in HeLa cells at different PROTAC degrader concentrations and different treatment times by western blot analysis. As shown in Fig. 2b, c, the degradation of BRD4 correlates with the concentration of ZXH-3-26 linearly within a certain range (nanomolar level), and the degradation of BRD4 became more significant. But the degradation efficiency decreased beyond a certain concentration due to the formation of binary complexes, which was called the “hook effect”. BRD4 degradation was determined using 100 nM ZXH-3-26. Obvious degradation of BRD4 was observed after 30 min, and complete depletion was observed after 4 h (Fig. 2e).

As a negative control, we used a BET inhibitor JQ1 during the treatment, which can only bind to the BD1/2 domain while not degrading BRD4 in HeLa cells (Supplementary Fig. S1f). Next, immunofluorescence detection of HeLa cells treated with different concentrations and different time points of ZXH-3-26 was conducted to characterize changes in BRD4 condensates, including BRD4 l and BRD4 s condensates. We found the reduction of BRD4 condensates in number, which was positively correlated with the western blotting results (Fig. 2c, d, f, g). Collectively, our findings demonstrated that PROTACs can be used as a suitable tool to disturb BRD4 condensates within cells and it should be employed in the study of the kinetics and molecular mechanisms of BRD4 phase separation.

### Perturbation of BRD4 condensates utilizing the mechanism of PROTACs

To further explore the mechanism of PROTACs on intracellular BRD4 condensates, we used chemical genetic and gene-editing approaches to investigate the changes in BRD4 condensates by manipulating proteasome function, CRBN-binding activity, and BRD4-binding activity. First, our results confirmed that JQ1 could not degrade BRD4. Moreover, the BRD4 condensates increased slightly under JQ1 treatment, which may be caused by the compensation effect following BRD4 inhibition, reflecting the advantages of PROTACs for studying phase separation (Supplementary Fig. S1g). Furthermore, the ability of ZXH-3-26 to degrade BRD4 was rescued with pretreatment of the proteasome inhibitor carfilzomib (Carf), JQ1, and pomalidomide (Poma), as well as recovering the BRD4 condensates (Supplementary Fig. S2a, b). Next, we established a *CRBN*-deficient HeLa cell line (HeLa-*CRBN*^−/−^) to illustrate the necessity of CRBN for ZXH-3-26-induced degradation. Whereas treatment of wild-type HeLa cells with ZXH-3-26 promoted degradation of BRD4, treatment of HeLa-*CRBN*^−/−^ cells with ZXH-3-26 was ineffective (Supplementary Fig. S2c). Interestingly, without CRBN, ZXH-3-26 could not induce BRD4 degradation and could only occupy the BRD4 active pockets like JQ1, resulting in ZXH-3-26 not being able to reduce the number of BRD4 condensates in HeLa-*CRBN*^−/−^ cells and even upregulating BRD4 to some extent (Supplementary Fig. S2d, e). Taken together, our results indicated that the reduction of BRD4 condensates was caused by PROTAC mechanistic functions.

### The dynamic perturbation process of BRD4 condensates by ZXH-3-26

**Fig. 3:**
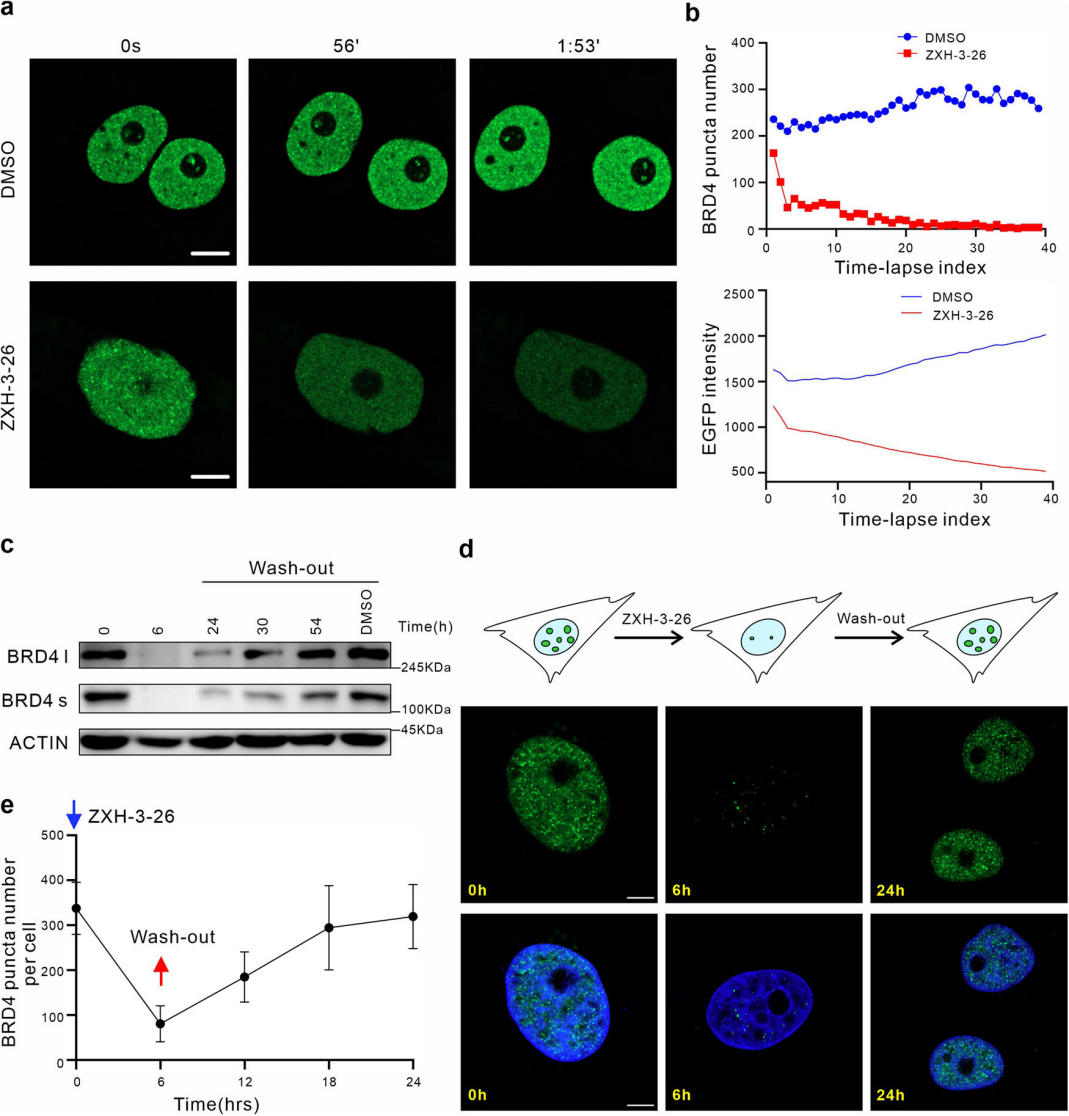
Determination of phase separation properties of BRD4 using ZXH-3-26 treatment. **a** Dynamic changes in BRD4 condensates with DMSO or ZXH-3-26 in EGFP-BRD4-expressing HeLa cells. Scale bars, 5 μM. **b** Statistical results of BRD4 condensate number and fluorescence intensity in EGFP-BRD4-expressing cells. **c** Western blotting was used to detect the protein level of BRD4 after the removal of ZXH-3-26 at different time points. **d** Immunofluorescence was used to track the BRD4 condensates after wash-out at different time points. Scale bars, 5 μM. **e** The number of BRD4 condensates per cell at different time points, *n* = 30–35 cells per time point.

Because the BRD4 degradation induced by ZXH-3-26 is fast and reversible, we hope to use this tool to explore the kinetic and molecular mechanisms of phase separation. For this reason, we constructed a HeLa cell line stably expressing EGFP-BRD4. EGFP-BRD4 fluorescence signal in the HeLa cells exhibited fast recovery after photobleaching (Supplementary Fig. S3a, b). As shown in time-lapse imaging, BRD4 condensates also underwent rapid fusion and fission in EGFP-BRD4-expressing HeLa cells^20^ (Supplementary Fig. S3c), demonstrating that BRD4 condensates possess properties of liquid-like condensates. To monitor the changes in BRD4 condensates during ZXH-3-26 treatment, a time-lapse image of EGFP-BRD4-expressing HeLa cells was performed. Our results showed that the number of BRD4 condensates in the nucleus gradually decreased within 2 h of ZXH-3-26 treatment, and the fluorescence signal also gradually weakened, which is consistent with our previous results of endogenous immunofluorescence (Fig. 3a, b; Supplementary Videos S1, S2).

To evaluate the effect of the reversibility of protein degradation of PROTACs on LLPS, the wash-out experiment of PROTACs was carried out. After treating HeLa cells with ZXH-3-26 for 6 h, ZXH-3-26 was washed-out and replaced with a fresh medium to observe the changes in BRD4 protein expression and condensates. Surprisingly, after performing the wash-out experiment, BRD4 condensates recovered rapidly and completely within 18 h (Fig. 3d, e). However, the protein level of BRD4 was only partially recovered after 18 h by western blot analysis (Fig. 3c). It is noteworthy to point out that the condensates are preferentially recovered during the gradual recovery of BRD4 protein in cells, which was discovered in phase separation using PROTACs for the first time.

### Modulated biological effects of phase separation by using ZXH-3-26

**Fig. 4:**
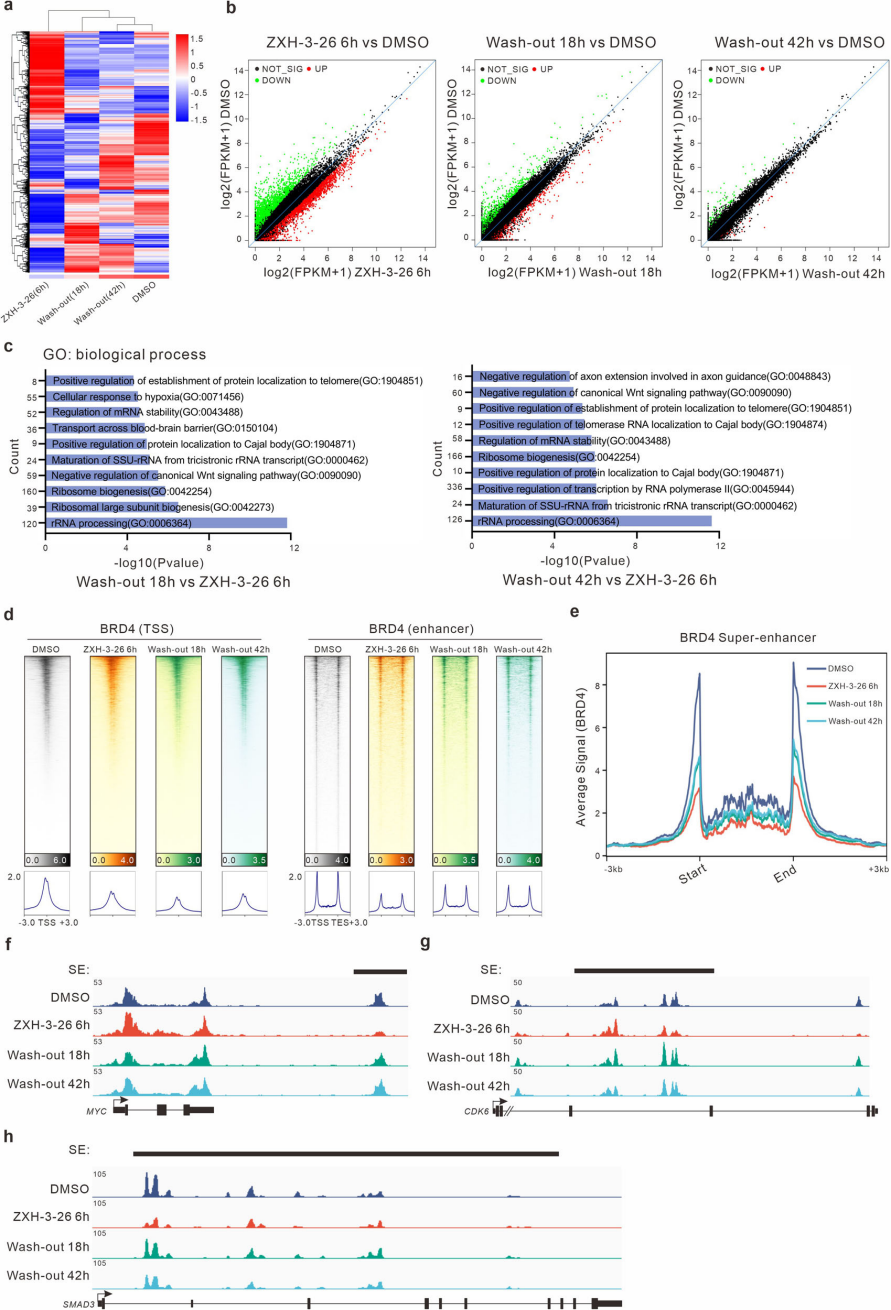
The changes in the cellular genome and chromatin state caused by ZXH-3-26 treatment and wash-out experiments. **a** The heat map of gene expression signatures of HeLa cells treated with ZXH-3-26 or wash-out experiments at different time points. **b** Compared with the DMSO group, the scatter diagram of gene changes after treatment with ZXH-3-26 for 6 h, wash-out 18 h, and wash-out 42 h. **c** GO analysis of significant genes at BRD4 degradation for 6 h and at different time points of wash-out experiments. **d** Heat map of BRD4 levels at transcriptional start sites (TSS) and enhancer after treatment with 6 h ZXH-3-26 (red), wash-out 18 h (green), wash-out 42 h (blue), or DMSO as vehicle control (gray). Each row shows ±3 kb centered on the BRD4 peak. Ranked was based on DMSO. CUT&Tag-seq signal was color-scaled intensities and normalized by spike-in controls. **e** Changes in BRD4 binding in SE regions were analyzed by CUT&Tag-Seq. **f**, **g**, **h** The gene track of BRD4 in the SE-driven locus of *MYC* (**f**), *CDK6* (**g**), and *SMAD3* (**h**) after wash-out experiments.

Previously, it was difficult to investigate the function of condensates in LLPS in a fast and reversible manner. To further elucidate the priority of BRD4 condensates over protein-level recovery, we used genome-wide measurements of chromatin structure and RNA transcriptome sequencing. First, RNA sequencing (RNA-seq) was performed on HeLa cells treated with PROTACs before and after the wash-out experiment. The results of the cluster analysis of the gene expression showed that cells after 42 h of wash-out experiments were closer to the control group treated with DMSO (Fig. 4a). With the increase in wash-out time, the different genes compared with the DMSO group decreased significantly (Fig. 4b). That is, BRD4 protein gradually recovered after PROTACs were washed-out, and BRD4 function gradually returned. GO enrichment analysis was performed on the RNA-seq results of the wash-out 18-h and 42-h group and the ZXH-3-26 degraded 6-h group. We found that the upregulated genes after the wash-out experiment were mainly correlated with the transcriptional regulation of RNA polymerase (Fig. 4c). For further validation, the CUT&Tag-seq was performed at different time points before and after wash-out experiments. The results showed that BRD4 was enriched in the enhancer regions, especially the SE regions after ZXH-3-26 depletion (Fig. 4d, e). We observed that BRD4 binding in the promoter and SE regions also had a slight rebound in the short time after removing ZXH-3-26 (Supplementary Fig. S4a, b). Meanwhile, the enrichment level of BRD4 in the promoter regions still decreased when the wash-out experiment continued for 18 h (Fig. 4d). It suggests that BRD4 condensates first recovered in the SE regions after wash-out. This is the first time that we can observe changes in the transcriptional regulation of biological macromolecular condensates using ZXH-3-26.

Several canonical BRD4 target genes are of interest. BRD4 binding at the SE regions of the *MYC* gene was reduced after ZXH-3-26 treatment (Fig. 4f)^37^. *CDK6*, another BRD4 target gene, restored BRD4 binding in SE regions after ZXH-3-26 depletion^38^ (Fig. 4g). SMAD3, a transcription factor that plays a vital role in TGF-β-induced growth inhibition^39^, also recovered BRD4-binding in the SE regions (Fig. 4h). In general, CUT&Tag-seq further confirmed what we observed: that is, during the gradual recovery of BRD4 protein by washing out PROTACs, BRD4 condensates recovered in a short time while preferentially occupying the SEs and further performing functions. These results were observed for the first time using PROTACs, which showed the advantages of the PROTAC in a rapid and reversible disturbance of phase separation system.

### ZXH-3-26-induced BRD4 degradation influenced changes in other components in condensates

**Fig. 5:**
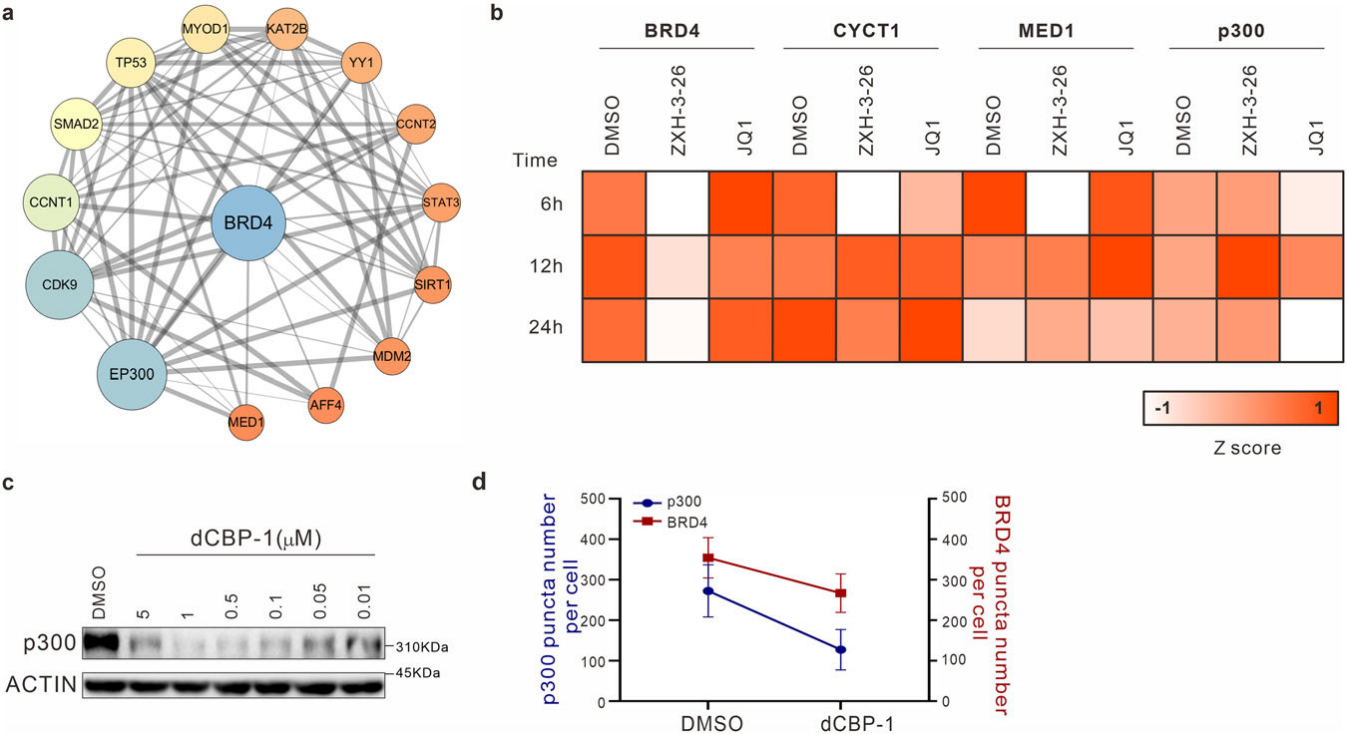
Using ZXH-3-26 to explore whether BRD4 is a key driver in condensates. **a** The network of BRD4 protein interaction. **b** Heat map of changes in the number of BRD4, CYCT1, MED1, and p300 condensates after ZXH-3-26 (100 nM) or JQ1 (500 nM) treatment for different times, *n* = 25–30 cells per time point. Z score was normalized by the formula, Z score = (X–mean)/SD, X was condensate number. **c** Western blotting of p300 in HeLa cells treated with different concentrations of dCBP-1 for 6 h. **d** Changes in the number of p300 or BRD4 condensates after dCBP-1 (1 μM) treatment for 6 h, *n* = 25–30 cells per point.

As described above, PROTACs can quickly downregulate BRD4 to regulate its condensates. Moreover, SEs can drive the expression of specific genes, and play key roles in stem cells and cancer cells^40^. Condensates at the SEs contain many transcription factors, and co-activators such as BRD4, MED1, p300, p-TEFb, and RNA Pol II^32,33,41,42^. Meanwhile, we showed the interaction network of BRD4-related proteins (Fig. 5a). It has been reported that MED1, p-TEFb, and p300 exhibited properties of forming condensates^10,11,34,43,44^. Therefore, we further applied the degrader ZXH-3-26 as a probe molecule to explore whether BRD4 perturbation is a key factor in the SE complex, and to observe the influence on other components in the SE complex. So, we conducted the degradation of BRD4 with ZXH-3-26 on HeLa cells and observed the above-mentioned related condensates.

After 6 h of ZXH-3-26 treatment, the condensates of cycling T1 (CYCT1) and MED1 also decreased in parallel with the reduction of BRD4 condensates (Fig. 5b), which was not observed in the treatment of inhibitor JQ1. With the prolonged treatment of the PROTACs (12 h and 24 h), however, the condensates of CYCT1 and MED1 gradually recovered and were no longer different from the control group (Fig. 5b). Meanwhile, as the upstream molecule of BRD4, p300 has no obvious perturbation in its condensates, which indicates that BRD4 in p300 condensate may not be a key protein. To this end, we used the p300 degrader dCBP-1^45^ to find that degradation of p300 can regulate BRD4 condensates, confirming that BRD4 is a directional dependence protein in p300 condensates (Fig. 5c, d). This is the first time that we observed when using PROTACs to confirm whether BRD4 is a key component in the other condensates of components in the SE regions. We inferred that ZXH-3-26 disrupted the BRD4–CYCT1–MED1 condensates at the SEs for a short period, but over time, cells initiated compensation effects to form new condensates without BRD4 to promote function effect.

**Fig. 6:**
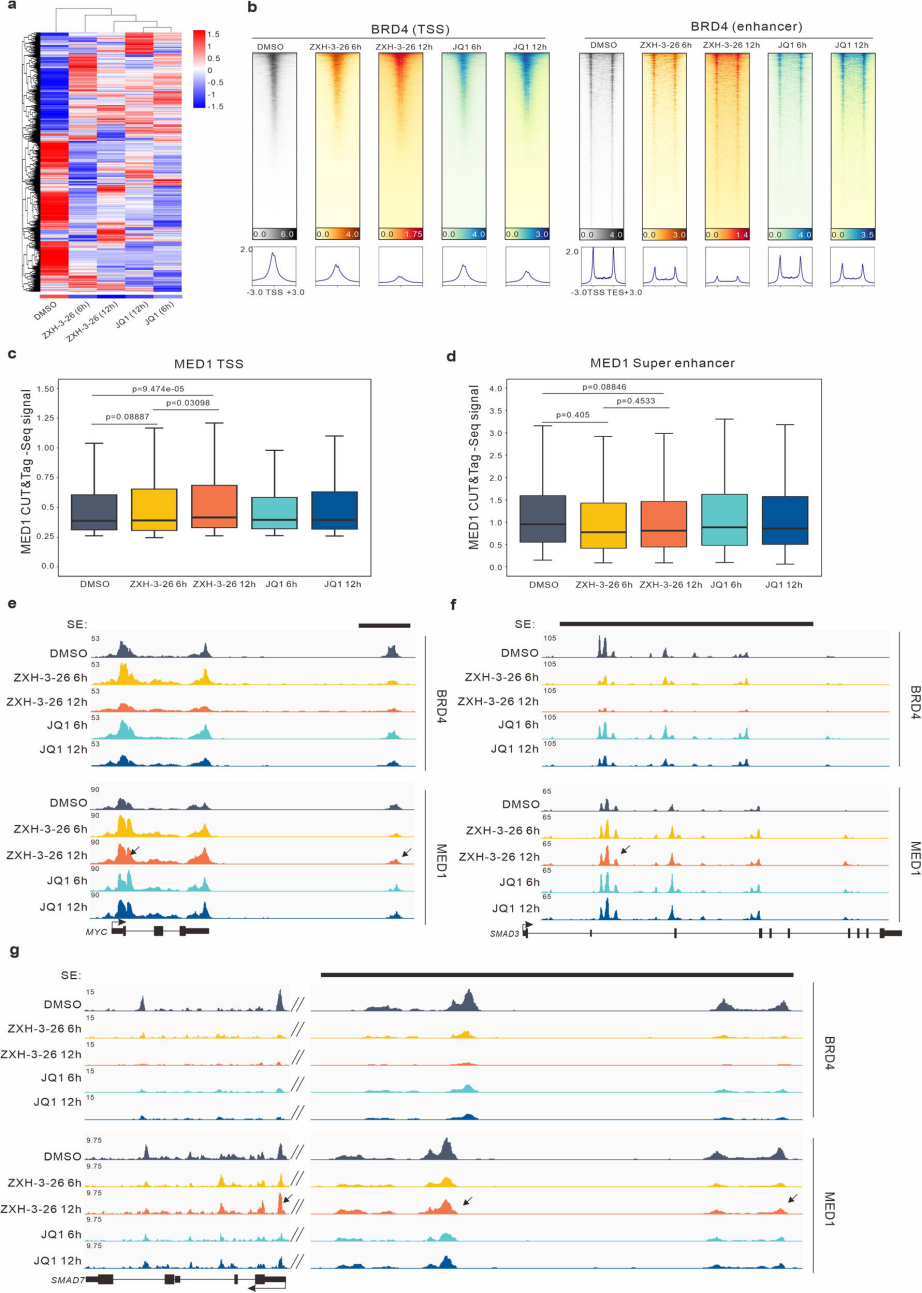
Effects of BRD4 degraders and BET inhibitors on BRD4, MED1 binding site enrichment. **a** Clustering heat map of changes in gene expression characteristics after 500 nM JQ1 or 100 nM ZXH-3-26 and DMSO treatment for 6 h or 12 h. **b** Heat map of BRD4-binding signal at the TSS and enhancer after treatment with 500 nM JQ1, 100 nM ZXH-3-26 for 6 h or 12 h or DMSO. **c** Enriched binding signal of MED1 at the TSS after treatment with 500 nM JQ1, 100 nM ZXH-3-26 for 6 h or 12 h or DMSO. **d** Enriched binding signal of MED1 at the SE regions after treatment with 500 nM JQ1, 100 nM ZXH-3-26 for 6 h or 12 h or DMSO. **e** The gene track of BRD4 and MED1 in the promoter- and SE-driven locus (*MYC* gene) after different time treatments with 500 nM JQ1 or 100 nM ZXH-3-26 for 6 h or 12 h or DMSO. **f**, **g** The gene track of BRD4 and MED1 in the promoter- and SE-driven locus of *SMAD3* (**f**), and *SMAD7* (**g**) after treatment with 500 nM JQ1, 100 nM ZXH-3-26 for 6 h or 12 h or DMSO.

To further confirm this hypothesis, we performed a multi-omics analysis under ZXH-3-26 treatment. First, we performed RNA-seq on HeLa cells treated with ZXH-3-26 and JQ1 for 6 h and 12 h. The results indicated that targeting BRD4 for degradation had a much stronger effect than inhibition (Fig. 6a). To investigate the short-term perturbation of components in SEs condensates by BRD4 degradation, we performed CUT&Tag-seq targeting BRD4 and MED1 in HeLa cells. After 6 h and 12 h of treatment with ZXH-3-26, the binding of BRD4 was significantly decreased in the promoter and enhancer regions compared with JQ1 (Fig. 6b), which further highlighted the advantages of degraders which were more rapidly perturbed than small molecule inhibitors in enhancer regions. The binding level of MED1 was higher at 12 h of ZXH-3-26 treatment than at 6 h of treatment in promoter regions (Fig. 6c). On the contrary, there was no significant difference in the SE regions (Fig. 6d). This may account for why MED1 condensates recovered to a similar level as the DMSO group at 12 h.

At the *MYC* SE regions, BRD4 binding was significantly reduced after treatment with ZXH-3-26, whereas JQ1 was ineffective. The enrichment of MED1 increased slightly after ZXH-3-26 treatment for 12 h. (Fig. 6e). Meanwhile, we found that the *MYC* promoter regions were more susceptible to regulation after ZXH-3-26 treatment for 12 h, and *SMAD3* and *SMAD7* had a similar situation (Fig. 6f, g). We reasoned that the observed phenomena were possibly due to the compensatory effect of other BET family members after BRD4 deletion to make MED1 proteins active in the promoter regions. Therefore, we used the pan-BET degrader dBET6 which can degrade BRD4 as well as BRD2 and BRD3^46^ (Supplementary Fig. S5a). Our results showed that after the treatment of 200 nM dBET6 for 6 h and 12 h, the MED1 and CYCT1 condensates were still in a downward trend compared with the control group (Supplementary Fig. S5b, c), and did not recover as the selective BRD4 degrader ZXH-3-26 did. When BRD4 is depleted, BRD2 or BRD3 may be able to form the complements. Therefore, we further carried out the co-localization assay of BRD3 and MED1. It has been reported that BRD3 and lncRNA DIGIT can form phase-separated condensates and promote transcription^47^. Meanwhile, BRD2 deletion increases BRD3 binding on promoters^48^. As expected, BRD3 condensates were detected in the MED1 foci (Supplementary Fig. S5d, e). The proportion of MED1 condensates co-localization with BRD3 condensates increased significantly after ZXH-3-26 treatment for 12 h (Supplementary Fig. S5f). Taken together, these data indicate that BRD3 plays a compensatory function in phase separation in cancer cells using ZXH-3-26.

## Discussion

PROTAC presents a new powerful method for studying protein–protein interactions and protein spatiotemporal dynamic regulation in cells because of their unique ability to rapidly degrade target proteins. Our study showed the advantages of PROTACs over inhibitors in the study of biomolecular condensates. After the addition of ZXH-3-26, the level of BRD4 protein decreased rapidly in a short time, and the condensates of BRD4 also decreased. Controlling the concentration of BRD4 protein can significantly regulate the formation of condensates in cells. Relative quantification of the correlation of endogenous BRD4 protein levels in cells with their condensates by BRD4-selective degraders, allows PROTACs to serve as an efficient probe to investigate the roles of BRD4 in phase separation (Supplementary Fig. S6).

Due to the reversibility of degraders, we observed for the first time that condensates preferentially recovered rapidly during the protein-level recovery of BRD4 after wash-out of PROTACs. At the same time, condensates will preferentially aggregate at SE regions, and then guide subsequent biological functions. In the future, we need to further study the differences in enhancer binding dynamics and the correlation between rapidly upregulated target genes and SE in the process of BRD4 recovery.

The fast and reversible nature of PROTAC allows us to study the phenotypes that cannot be observed by genetic tools such as CRISPR-Cas9 or RNAi, without being affected by compensatory effects after genetic manipulation, enabling dynamic monitoring over short periods^49^. For instance, treatment of ZXH-3-26 enabled us to observe the reduction of other condensates MED1 and CYCT1 at the SE regions for a short time. BRD4 interacts with MED1, and BRD4 recruits the p-TEFb complex into the transcription initiation phase. In the case of acute degradation of BRD4, its associated condensates are also destroyed to a certain extent, but for p300, as the upstream molecule of BRD4, its condensates are not disturbed. Under the continuous degradation of BRD4, the MED1 and CYCT1 condensates will recover to the same level as the control group. Our study further suggests that other members of the BET family, BRD3 played a compensatory mechanism, which was observed for the first time in phase separation (Fig. 1).

In conclusion, our research showed that BRD4 small molecule degraders can serve as effective tools to study BRD4 in phase separation, providing a new perspective for dissecting the molecular mechanisms and dynamics of BRD4 in phase separation. We believe that degraders can provide unique perspectives and opportunities for exploring the interaction of condensates under pathology and optimizing drug design in the near future.

## Materials and methods

### Cell line culturing and treatment

All cell lines were tested for mycoplasma contamination according to the manufacturer’s guide (Mycoblue Mycoplasma Detector, D101-01). The HeLa cell line was obtained from ATCC. The EGFP-BRD4 HeLa cell line was kindly provided by Dr. Wei Wu from the School of Life Sciences (Tsinghua University, Beijing, China). HeLa and EGFP-BRD4 HeLa cells were maintained in DMEM (Gibco) supplemented with 10% FBS (BI) and 1% Penicillin-Streptomycin. All cell lines were cultured at 37 °C with 5% CO_2_. ZXH-3-26 was purchased from MCE. JQ1, pomalidomide, and Carfilzomib were purchased from Selleck. dBET6 was purchased from TargetMol. dCBP-1 was synthesized as described^45^. All treatments were performed under a complete medium. Compounds were dissolved in DMSO for storage (10 mM). Next, the compounds were diluted in the culture medium and added to each well to the indicated concentration. The final DMSO concentration in the culture medium was no more than 0.5%.

### Immunofluorescence

Cells were cultured on coverslips and fixed with 4% paraformaldehyde (PFA) for 15 min at room temperature. Cells were permeated by PBS supplemented with 0.2% Triton X-100 for 10 min at room temperature and blocked by PBS supplemented with 5% BSA and 0.2% Triton X-100 for 60 min at room temperature. The following primary antibodies were used: anti-BRD4 (Abcam, ab128874, 1:200 dilution), anti-MED1 (Abcam, ab64965, 1:200 dilution), anti-p300 (Abcam, ab259330, 1:200 dilution), anti-Cyclin T1 (Abcam, ab184703, 1:200 dilution). Antibodies were diluted in a blocking buffer and incubated overnight at 4°C. After washing three times with PBS for 5 min each. Fluorescent secondary antibody (ABclonal, AS011, 1:500 dilution) was incubated by PBS supplemented with 0.2% Triton X-100 for 60 min at room temperature in dark. The cells were washed with PBS three times for 5 min each. 4,6-diamidino-2-phenylindole (DAPI) (Beyotime, C1005) was used to stain nuclei for 5 min at room temperature in the dark. Coverslips were mounted onto microslides (CITOTEST, 1A5101). Coverslips were sealed with transparent nail polish and stored at −20 °C. Images were acquired at Zeiss LSM980 Airyscan2. Images were processed and analyzed using Imaris v9.9.

### FRAP

The EGFP-BRD4 Hela cells were grown on matrigel-treated glass-bottom confocal petri dishes (Cellvis). The Nikon A1 HD25 confocal laser scanning microscope was used for photobleaching. Images were acquired using a 100× oil-immersion objective, and the microscope was controlled using NIS-Elements v5.2. For FRAP of BRD4 condensates, bleaching was performed with a 488 nm laser at 100% power with 3 frames being acquired before bleaching. Fluorescence recovery was recorded every 0.25 s for 30 s after bleaching. Analyses of the fluorescence intensity of the bleaching region were carried out using the NIS-Elements v5.2.

### Construction of *CRBN*^−/−^ HeLa cells

*CRBN* sgRNA oligos were cloned into the px458 vector expressing Cas9 and EGFP (Addgene #48138). The sequence was 5′- TAAACAGACATGGCCGGCGA-3′. HeLa cells were sorted EGFP-positive cells after transfection 48 h. The sorted cells were seeded in 96-well plates until cell clones were ready to be picked. The clones were confirmed by DNA sequencing and Western blot analysis.

### Live-cell imaging

Cells were grown on matrigel-treated glass-bottom confocal petri dishes (Cellvis) and before imaging cell culture media was replaced with ZXH-3-26 culture media and imaged using Nikon A1 HD25 confocal laser scanning microscope. Cells were imaged on a heated stage (37 °C) and supplemented with 5% CO_2_. Additionally, the microscope was placed in an incubation chamber heated to 37 °C. Dynamic degradation images were acquired at intervals of 3 min and were analyzed with NIS-Elements v5.2.

### Western blotting and protein degradation assay

Cells were cultured under different compound treatment conditions. Cells were seeded in 6-well or 12-well plates. After treatment, cells were collected and washed with PBS, then lysed in RIPA lysis buffer containing Protease Cocktail (Yeasen, 20123ES) and PMSF (Beyotime) for 30 min, then added 2× loading buffer (Beyotime) containing mercaptoethanol. Then, samples were heated in a 100 °C dry bath for 10 min. Cell lysates were added to 8%–10% SDS-PAGE gels and transferred to the PVDF membrane (Merck Millipore). After transfer, the membrane was blocked with 5% non-fat milk in TBST for 1 h at room temperature and shaking. The membrane was incubated with 1:1000 anti-BRD4 (Abcam, ab128874), anti-BRD2 (Abcam, ab243865), anti-BRD3 (Santa Cruz, sc-81202), and anti-β-Actin (ABclonal, AC026) antibody diluted in the corresponding antibody dilution buffer (Beyotime) and incubated overnight at 4 °C, with shaking. Then, the membrane was incubated with 1:10,000 appropriate HRP-conjugated secondary antibodies for 1 h at room temperature and washed five times in TBST for 5 min. Membranes were developed with enhanced chemiluminescence (DiNing ECL Enhanced) and imaged using a Tanon 5200 luminous.

Protein degradation was assessed by grayscale analysis. The grayscale analysis was conducted by ImageJ software. Statistical analysis was carried out using GraphPad Prism v.8.

### Analysis of apoptotic cells by flow cytometry

For each sample, apoptosis was evaluated by Annexin V-fluorescein isothiocyanate (FITC) and propidium iodide (PI) apoptosis detection kit (Solarbio, CA1020) according to the manufacturer’s instructions^50^. Cells were then sorted on a CytoFlex LX and analyzed using FlowJo V10 software.

### Protein–protein interaction network

A composite protein–protein interaction network was built by combining STRING^51^. Only physical protein–protein interactions were considered, and the interactome was filtered by edge confidence scores. Statistical analysis was carried out using Cytoscape v3.7.1.

### CUT&Tag-Seq data processing and analysis

The Hyperactive Universal CUT&Tag Assay Kit for Illumina (Vazyme, TD903-01) was used for the test. CUT&Tag sequencing data processing was performed as previously described^52^. Briefly, paired-end reads were aligned to GRCh38p13 (https://ftp.ebi.ac.uk/pub/databases/gencode/Gencode_human/release_40/GRCh38.p13.genome.fa.gz) using bowtie2 (v2.4.1) with the options “--local --very-sensitive --no-unal --no-mixed --no-discordant --phred33 -I 10 -X 700”. Model-based Analysis of ChIP-Seq (MACS2 v2.2.6) was used for peak calling with parameters “macs2 call-peak -t input_file -p 1e-5 -f BAMPE --keep-dup all -n output_file_name –outdir output_directory”.

Bigwig file, which is used for the display of dense, continuous data as a graph, was generated by the bamCoverage command in deepTools (v3.3.0)^53^ software, during which the CPM normalization method was involved. And, IGV (IGV: Integrative Genomics Viewer) was used for visualization^54^.

SE regions were identified using ROSE software developed by Richard A. Young^41,55^.

### RNA-seq data processing and analysis

3 ng of total RNA were used to create a library for transcriptome Sequencing on an Illumina NovaSeq 6000 platform (Tiangen). Reads were aligned to GRCh38p13 using RSEM (v1.3.1)^56^ with options “--bowtie2 --bowtie2-sensitivity-level fast”. FPKM (Fragments Per Kilobase of exon model per Million mapped fragments) was then calculated for each gene.

## Supplementary information

Supplementary materials

Supplementary Video S1

Supplementary Video S2

## Data Availability

All sequencing data have been uploaded to the Genome Sequence Archive in the National Genomics Data Center, Beijing Institute of Genomics, Chinese Academy of Sciences, under accession number HRA002934.
