# Supplementary material for: BRD4-targeting PROTAC as a unique tool to study biomolecular condensates
Source: Cell Discov. 2023 May 9;9:47. doi: 10.1038/s41421-023-00544-0 (PMC10167318; doi:10.1038/s41421-023-00544-0)
Supplement: Supplementary file 1 — Supplementary materials [file 41421_2023_544_MOESM1_ESM.docx]

Supplementary Video S1 Dynamic changes in BRD4 condensates in EGFP-BRD4 expressing HeLa cells with DMSO.

Supplementary Video S2 Dynamic changes in BRD4 condensates in EGFP-BRD4 expressing HeLa cells with ZXH-3-26 treatment.

**
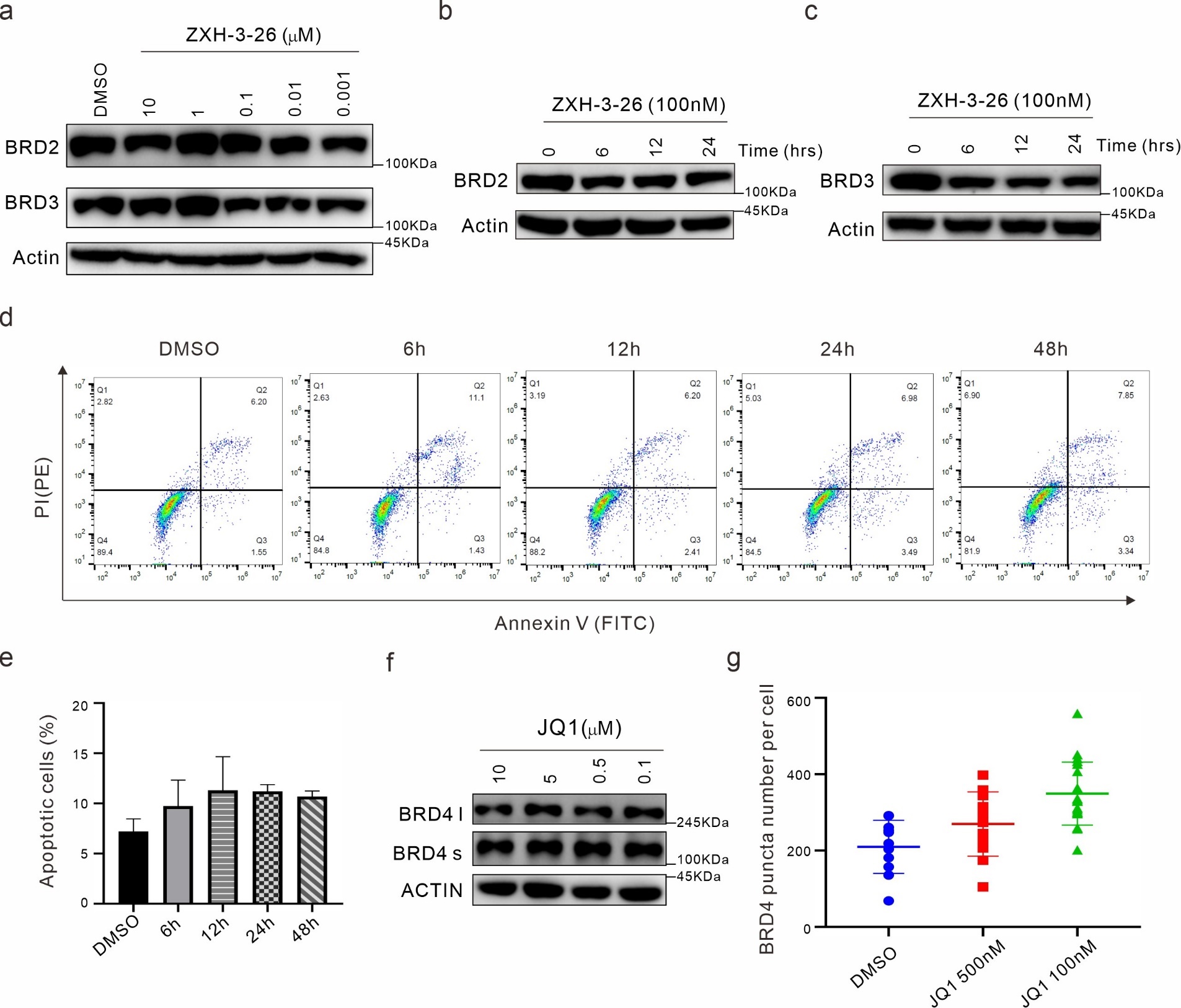
Supplementary Fig. S1 Multiple methods evaluating the efficacy and effects of ZXH-3-26 *in* *vitro*.** (a) Western blotting was performed to assess the protein levels of BRD2 and BRD3 in HeLa cells treated with different concentrations of ZXH-3-26 for 6 hours. (b) BRD2 was detected by western blotting at different time points after ZXH-3-26 treatment at 100 nM concentration. (c) BRD3 was detected by western blotting at different time points after ZXH-3-26 treatment at 100 nM concentration. (d) Cell apoptosis was shown by flow cytometry at different time points after ZXH-3-26 treatment at 100 nM concentration. (e) Cell apoptosis results were quantified. (f) The protein level of BRD4 in HeLa cells treated with different concentrations of JQ1 for 6 hours. (g) The number of BRD4 condensates in cells treated with different concentrations of JQ1 for 6 hours. n=20-30 cells per time point.

**
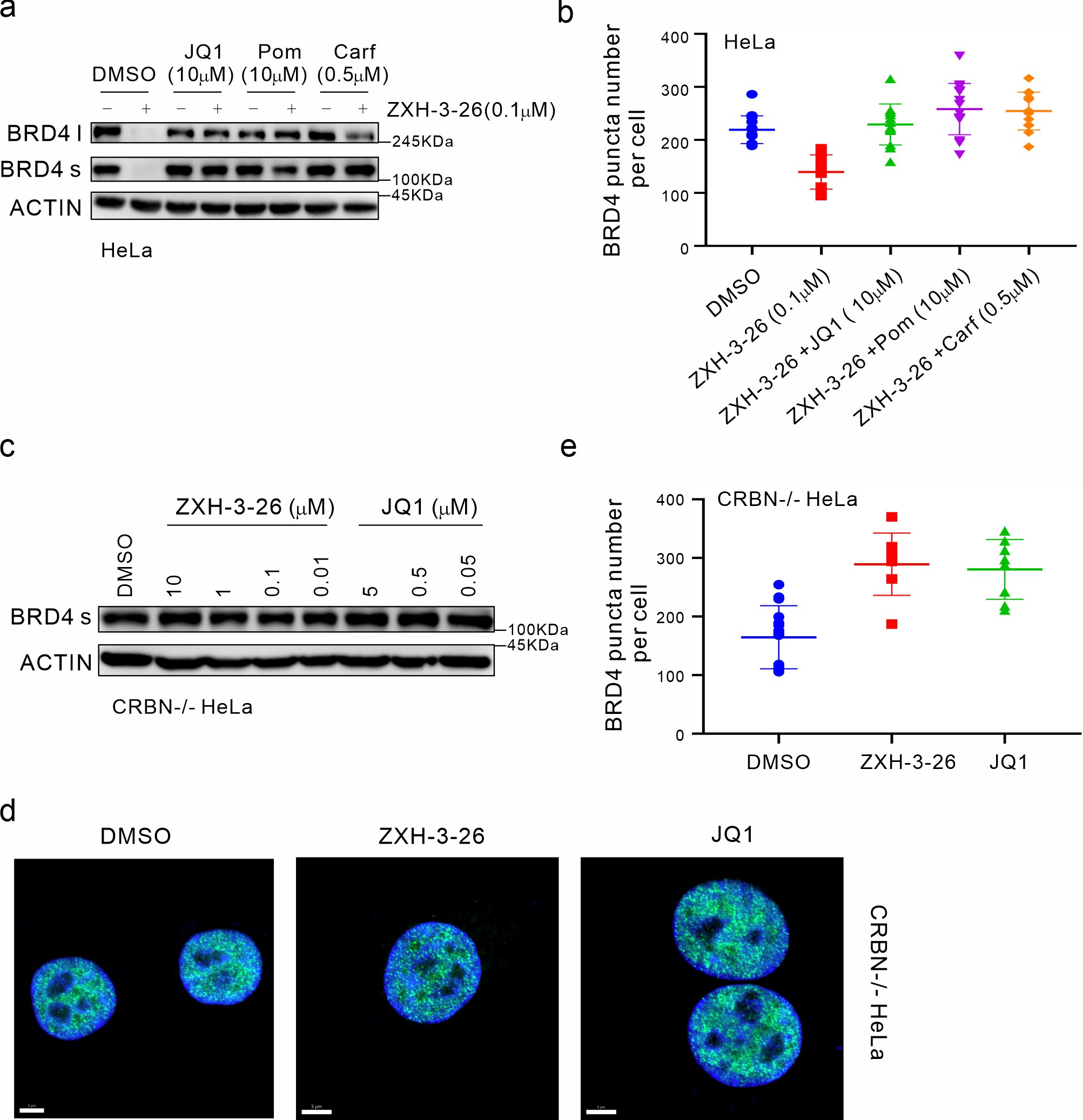
****Supplementary Fig. S2 ZXH-3-26 acts through the classical PROTAC degradation mechanism.** (a) HeLa cells were pretreated with JQ1, Poma, Carf for 2h and then ZXH-3-26 treatment for 6h. BRD4 was detected by western blotting. (b) The number of BRD4 condensates per cell treated with indicated small molecules. n=20-30 cells per time point. (c) Western blotting of BRD4 in CRBN-/- HeLa cells treated with different concentrations of ZXH-3-26 and JQ1 for 6 hours. (d) Immunofluorescence was used to track the BRD4 condensates after treatment with 100nM ZXH-3-26 and 500nM JQ1 for 6h. (e) The number of BRD4 condensates were quantified in (d). n=20-30 cells per time point.


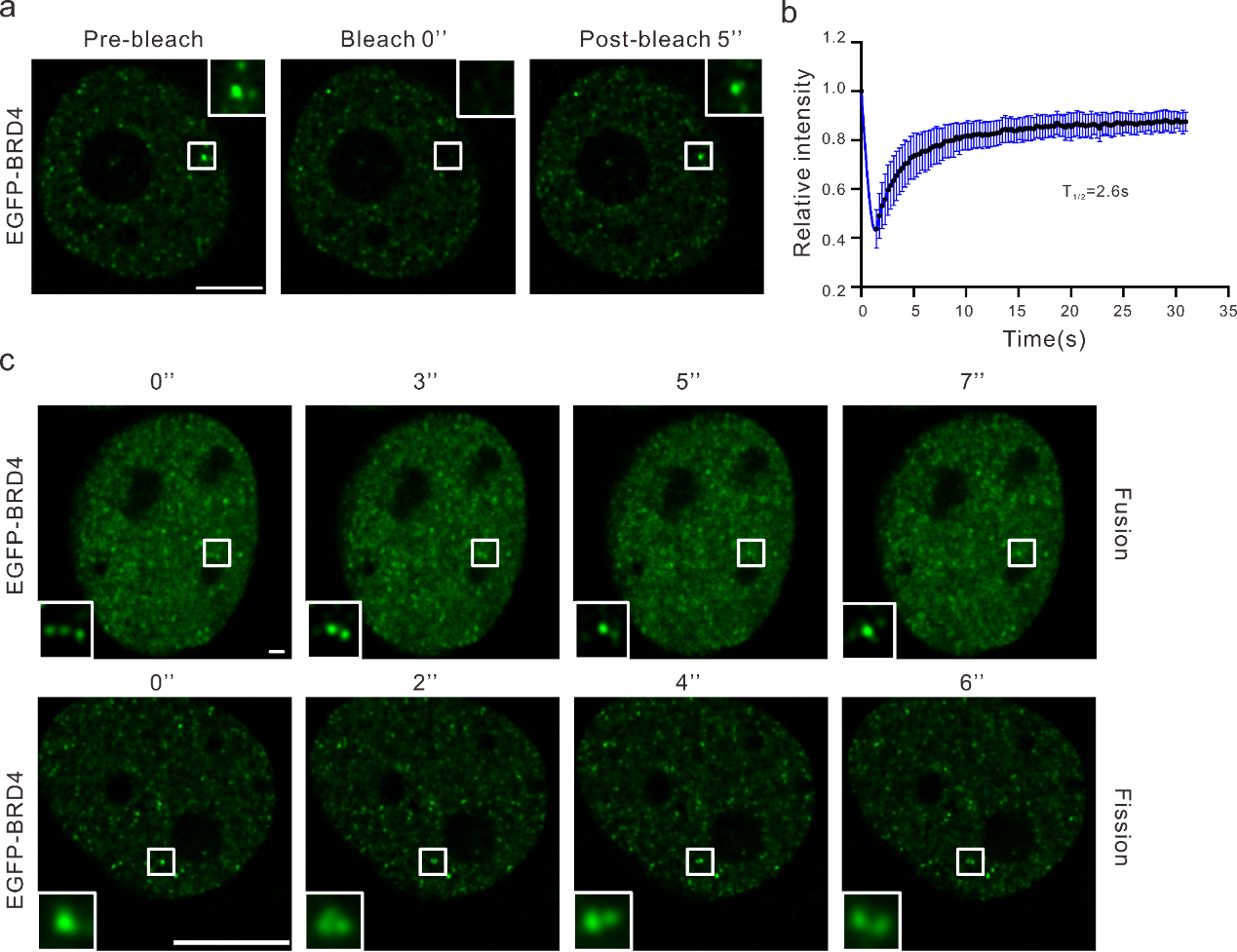


**Supplementary Fig. S3 EGFP-BRD4 condensates exhibit LLPS properties.** (a) Photo bleaching analysis of EGFP-BRD4 puncta in HeLa cells. (b) FRAP experiments were quantified. Results shown were from 25 replicates. (c) Time-lapse images of EGFP-BRD4 puncta in HeLa cells. BRD4 puncta fusion and fission were shown in highlighted box.

**
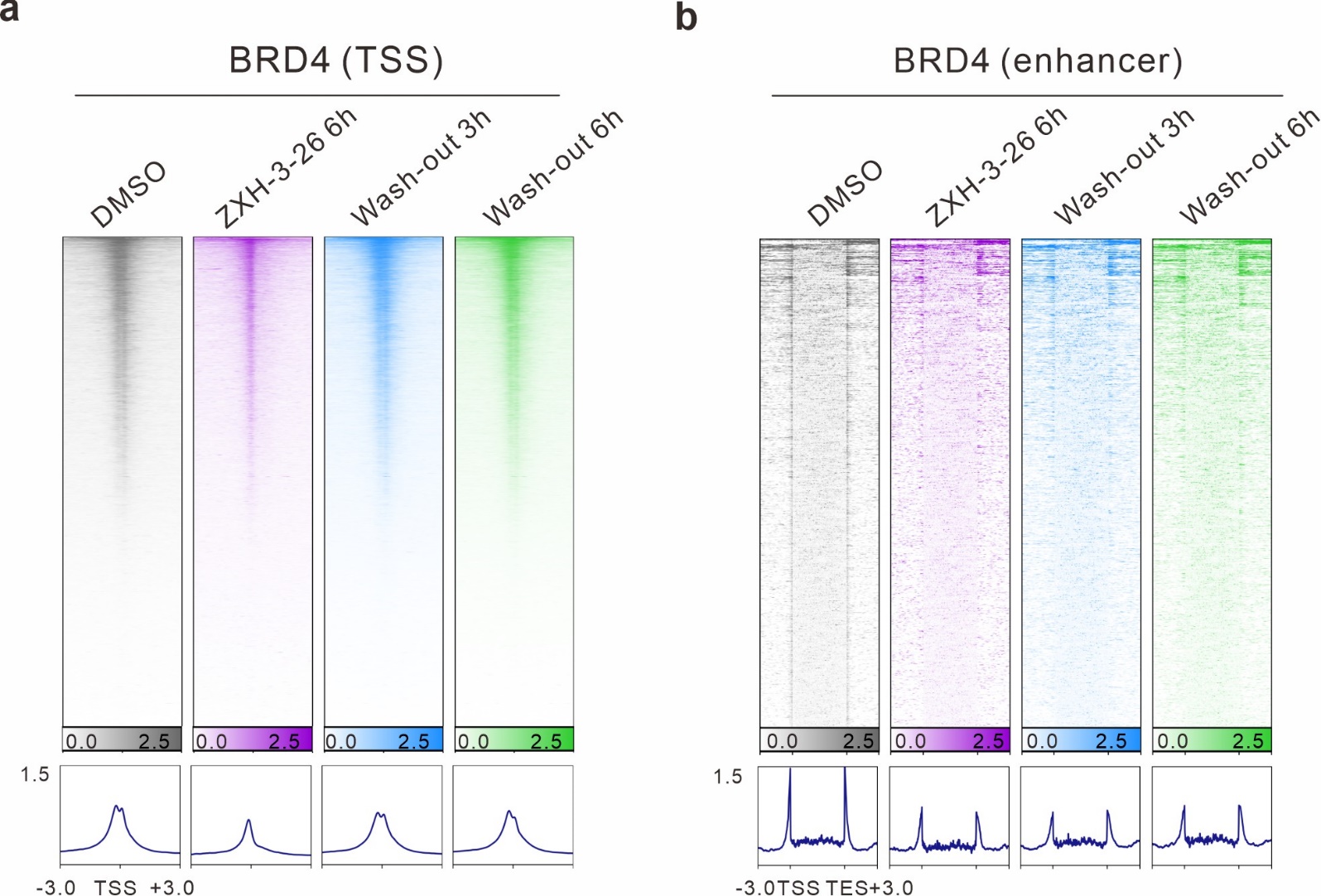
Supplementary Fig. S4** (a) Heatmap of BRD4 levels at transcriptional start sites (TSS) after treatment with 6h ZXH-3-26, wash-out 3h, wash-out 6h, or DMSO as vehicle control. Each row shows ± 3kb centered on the BRD4 peak. Ranked based on DMSO. CUT&Tag signal was color-scaled intensities and normalized by spike-in controls. (b) Same as in (a), but for enhancers.

**
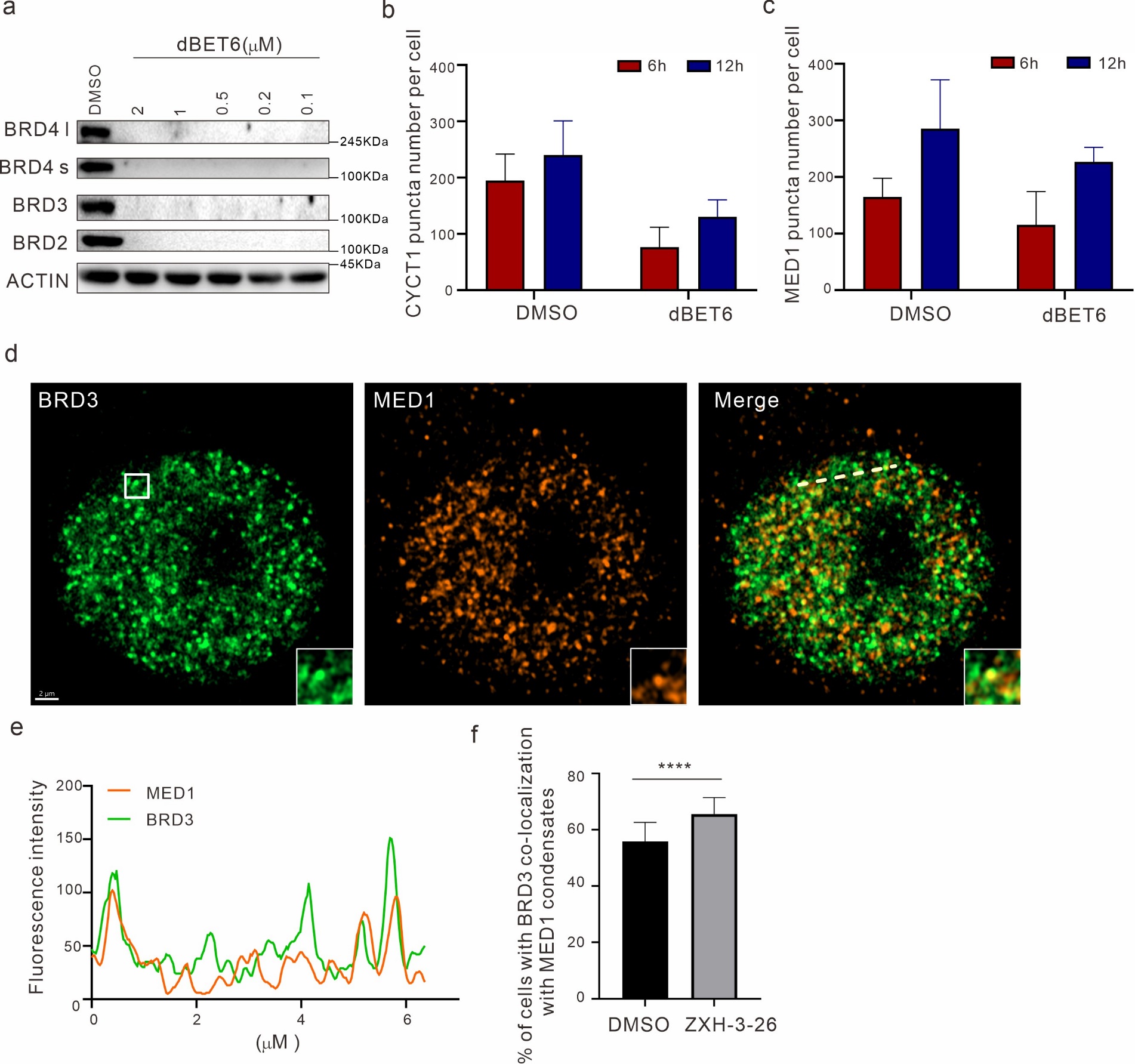
Supplementary Fig. S5** (a) Western blotting of BRD2, BRD3, and BRD4 in HeLa cells treated with different concentrations of dBET6 for 6 hours. (b) The number of CYCT1 condensates after 6 hours and 12hours of dBET6 (200 nM) treatment, n=25-30 cells per time point. (c) The number of MED1 condensates after 6 hours and 12hours of dBET6 (200 nM) treatment, n=25-30 cells per time point. (d) Representative images indicating the co-localization of BRD3 (green) with MED1 condensates (orange) in HeLa cells after ZXH-3-26 treatment for 12 hours. (e) Quantification of fluorescence intensity of MED1 and BRD3 along the line indicated in the merged image was shown. (f) Percentage of cells (n=75-80) containing BRD3 condensates co-localization with MED1 condensates. ****p<0.0001.

**
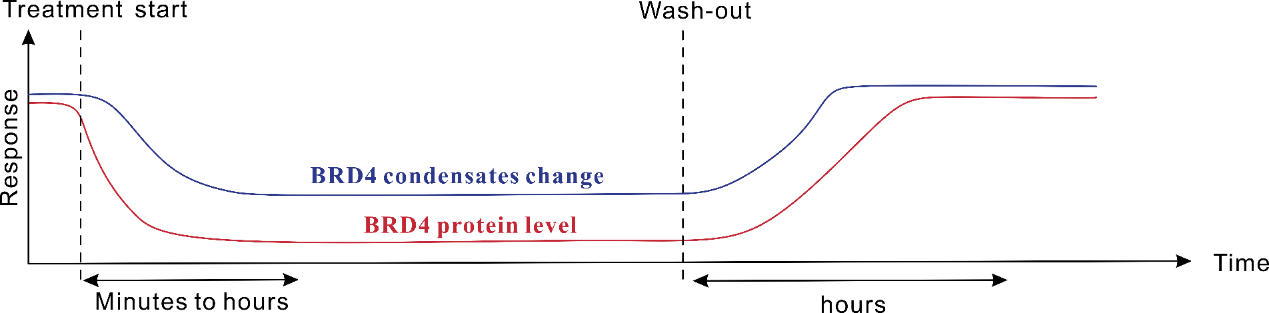
Supplementary Fig. S6 BRD4 degraders regulating BRD4 phase separation.**

The addition of the degrader rapidly disturbed the BRD4 protein level and BRD4 condensates, and the BRD4 condensates recovered preferentially from the protein level after the degrader wash-out.
